# A model-based selection of oral cancer patient for passive scattering proton beam therapy

**DOI:** 10.1093/jrr/rrag042

**Published:** 2026-06-04

**Authors:** Yuki Narita, Kanako Takayama, Takahiro Kato, Shinya Komori, Ichiro Seto, Tatsuhiko Nakasato, Masao Murakami

**Affiliations:** Department of Radiation Physics and Technology, Southern Tohoku Proton Therapy Center, 7-172 Yatsuyamada, Koriyama, Fukushima 963-8052, Japan; Department of Radiation Oncology, Southern Tohoku Proton Therapy Center, 7-172 Yatsuyamada, Koriyama, Fukushima 963-8052, Japan; Department of Radiation Physics and Technology, Southern Tohoku Proton Therapy Center, 7-172 Yatsuyamada, Koriyama, Fukushima 963-8052, Japan; Department of Radiological Sciences, School of Health Sciences, Fukushima Medical University, 10-6 Sakaemachi, Fukushima, Fukushima 960-8516, Japan; Department of Radiation Physics and Technology, Southern Tohoku Proton Therapy Center, 7-172 Yatsuyamada, Koriyama, Fukushima 963-8052, Japan; Department of Radiation Oncology, Southern Tohoku Proton Therapy Center, 7-172 Yatsuyamada, Koriyama, Fukushima 963-8052, Japan; Department of Radiation Oncology, Southern Tohoku Proton Therapy Center, 7-172 Yatsuyamada, Koriyama, Fukushima 963-8052, Japan; Department of Radiation Oncology, Southern Tohoku Proton Therapy Center, 7-172 Yatsuyamada, Koriyama, Fukushima 963-8052, Japan

**Keywords:** proton beam therapy, model-based approach, oral cancer

## Abstract

Background and purpose: To identify patients with oral cancer (OC) who benefit from proton therapy versus photon therapy using a model-based approach (MBA) to reduce late adverse events. Material/Methods: Passive scattering proton beam therapy (PSPT) and volumetric modulated arc therapy (VMAT) plans were established for 40 patients with OC, consisting of a dose of 70 Gy (relative biological effectiveness) in 35 fractions. At least 95% of the planning target volume received 95% of the prescribed dose, and the dose to the organs at risk was restricted to be as low as possible. Normal tissue complication probability (NTCP) was used to estimate the risk of late adverse events, including dysgeusia, xerostomia and osteoradionecrosis of the mandible. Patients who were considered as candidates for PSPT were selected if the NTCP difference between both plans (ΔNTCP) exceeded the threshold defined in the Dutch national indication protocol. Results: PSPT reduced the doses to the spinal cord, pharynx, oral cavity and contralateral salivary glands compared with VMAT. PSPT was also superior for the risk evaluation of each late adverse event calculated by incorporating dose metrics and patient baseline risk factors. For the MBA using ΔNTCP and the thresholds, PSPT was indicated in 24 of the 40 cases, and the remaining 16 cases were considered to be equivalent to VMAT. Conclusion: MBA was used to select OC cases that were candidates for PSPT. Compared with VMAT, the risk of expected late adverse events was significantly reduced with PSPT, and 60% of patients were considered eligible for PSPT.

## INTRODUCTION

Surgery is the standard curative treatment for oral cancer (OC); however, radiotherapy (RT) may be selected for cases in which surgery is not suitable or in advanced cases [1–3]. Chemotherapy is often used in combination, and simultaneous chemoradiotherapy with platinum agents has shown excellent therapeutic effects and has become the standard treatment for inoperable head and neck cancer [4, 5]. There is also significant interest in the combination of selective intra-arterial infusion chemotherapy (IAIC) for OC, which exhibits poor sensitivity to drugs and radiation [6–8].

Photon beams are often used in external RT for OC. Irradiation techniques, such as intensity-modulated RT and volumetric modulated arc therapy (VMAT), are recommended to reduce the dose to organs at risk (OARs) near the target, such as the spinal cord, salivary glands and mandible [9, 10]. Proton beam therapy (PBT) also represents an option for reducing OAR dose. For PBT, most of the energy is deposited at a specific depth (the Bragg peak) in the beam path [11, 12]. Theoretically, the superior beam characteristics enable higher doses to be administered, which may result in better local control, while maintaining radiation-induced adverse events within an acceptable range compared with photon beam therapy. Nonetheless, there is a lack of comparative evidence for the actual biological effects, such as tumor control and acute and late adverse events; thus, the efficacy of PBT for OC remains unclear. As with any new treatment regimen, sufficient consensus must be achieved by conducting randomized controlled trials (RCTs), which requires considerable time and expense. Moreover, technological developments in PBT are progressing rapidly. Therefore, by the time the results from RCT are available, they may represent outdated technology [13].

Recently, a model-based approach (MBA) has been developed to identify patients who are expected to benefit the most from PBT as an alternative approach [14, 15]. This is an evidence-based evaluation method for selecting patients for PBT who will experience reduced radiation-induced adverse events. The clinical benefits of PBT may be determined by estimating the potential risk of adverse events using the normal tissue complication probability (NTCP) for dose differences between RT techniques. This method is approved and accepted in Europe, primary in the Netherlands, as a basis for making treatment decisions [16]. It provides alternative or supplementary data in the absence of RCT results. In particular, MBA is expected to predict the effectiveness of PBT in rare cancers such as OC, for which it is difficult to collect a large number of cases for RCT. Therefore, in this study, we evaluated the effectiveness of PBT versus photon beam therapy for OC cases using MBA.

## MATERIALS AND METHODS

### Patient selection

A comparative planning study was performed using imaging data from OC patients who underwent PBT and IAIC at our institution between October 2009 and October 2020. The OC subsites included the tongue, hard palate, maxillary gingiva, mandibular gingiva, floor of the mouth and buccal mucosa. Our institutional review board approved the protocol for this retrospective analysis (No. 678).

### Delineation of target volumes and OARs

A thermoplastic mask was used to immobilize the head and neck, and computed tomography (CT) images were captured using a 1-mm slice thickness. Aquilion LB (Canon Medical Systems, Tochigi, Japan) was used as the CT scanner. Magnetic resonance imaging with a 3-mm slice thickness was conducted and registered to the CT images to delineate the target volume and OARs using the Focal treatment planning support system (Elekta, Stockholm, Sweden). Gross tumor volume (GTV) was delineated by a radiation oncologist using these images. Clinical target volume (CTV) was defined as the three-dimensional expansion of the GTV with a 3–7 mm margin to compensate for microscopic tumor extension and adjusted for each case. Planning target volume (PTV) was created with a 3-mm uniform margin on the CTV for photon therapy and evaluation of PBT. The spinal cord, pharynx, mandible, parotid glands, submandibular glands and sublingual glands surrounding the target were contoured as the OARs.

### Treatment planning

All PBT plans were generated retrospectively using the original planning CT data and were not used for clinical treatment delivery. The PBT plan was created based on the CT images using the XiO-M treatment planning system (version 4.34.02, Hitachi, Kashiwa, Japan). The proton beam irradiation was assumed using passive scattering PBT (PSPT) and the MELTHEA proton-type particle therapy system (Hitachi, Kashiwa, Japan). For PSPT planning, unlike photon beams, the PTV concept is different. No margin was uniformly added to the entire CTV circumference, and the margins placed on the CTV were mathematically calculated. When expanding the margins, the penumbra (7 mm) and setup uncertainties (3 mm), range uncertainties (3 mm) and Hounsfield unit uncertainties (3.5%) should be considered. The distal margin (DM), proximal margin (PM), lateral margin (LM) and compensator smear (CS) were calculated for each beam using the formulas proposed by Moyers *et al*. [17]. These values were adjusted according to the positional relationship between the target and the OARs. The average values for DM, PM, LM and CS were ~6, 3, 10 and 5 mm, respectively. The irradiation field was established using the multi-leaf collimator with a 5-mm leaf width built into the snout, which was placed near the patient for better dose distribution. The plan involved irradiation by three to four fields of the anterior and lateral beams. The beam angle was slightly adjusted to consider the target shape and dose to the skin. The wobbler and ridge filter methods, which are the passive scattering methods, were used to form the irradiation field. A pencil beam convolution algorithm was used for dose calculation during the treatment planning. The total prescription dose was 70.0 Gy relative biological effectiveness (RBE) in 35 fractions. A constant RBE of 1.1 was assumed. At least 95% of the PTV was expected to receive 95% of the prescribed dose (V_95%_ > 95%). A 2% dose of the PTV corresponded to <107% (D_2%_ < 107%). The dose constraints for the OARs were limited below the tolerance dose of each organ. In this study, dose constraints were primarily applied to spare the oral cavity, salivary glands and mandible to enable model-based evaluation of dysgeusia, xerostomia and osteoradionecrosis. The oral cavity, including the tongue, was preferentially spared. The maximum dose to the mandible was lower than 60 Gy (RBE), and the mean dose to the parotid, submandibular and sublingual glands was lower than 26 Gy (RBE). Additionally, to maintain consistency with our institutional clinical practice, the maximum dose to the spinal cord and pharynx was lower than 50 Gy (RBE) and 60 Gy (RBE), respectively. These dose constraints were based on the values ​​presented in the quantitative analyses of normal tissue effects in the clinic review [18] and applied within a range where the target coverage had not deteriorated. The photon irradiation method was assumed to be VMAT, and treatment planning was performed using the Eclipse treatment planning system (version 15.6, Varian Medical Systems, Palo Alto, USA) with an Acuros XB. Dose calculation was performed for the PTV under the conditions of one isocenter and two full rotations, with a 6 MV flattening-filter beam. Start and stop angles were slightly adapted to avoid unnecessary exposure of normal tissue. To assure comparability, the same dose prescription and constraints for the PTV and OARs, as in PSPT planning, were applied.

### Dose volume comparison and MBA

For dose volume evaluation, all treatment planning data were aggregated into the RayStation treatment planning system (version 4.7.4.4, RaySearch Laboratories, Stockholm, Sweden). The dose volume parameters between PSPT and VMAT plans were compared. Adverse events observed in RT for OC include mucositis, dermatitis, osteomyelitis, dysgeusia, xerostomia and osteoradionecrosis of the mandible. Of these, dysgeusia, xerostomia and osteoradionecrosis of the mandible were selected to predict the incidence of adverse events, as they significantly affect quality of life. Published NTCP models of these adverse events were selected, and models that predicted adverse events of at least grade II or higher in the common terminology criteria for adverse events score (version 5) were selected. Although the available NTCP models are limited, the following three previously reported models were evaluated: the NTCP model for grade II dysgeusia, xerostomia and grade IV osteoradionecrosis of the mandible were selected. The calculation formula for NTCP is as follows:


(1) \begin{equation*} {NTCP}_{dysgeusia}={\left(1+{\left(\frac{TD_{50}}{Oral\ cavity\ gEUD}\right)}^{4\times{\gamma}^{50}}\right)}^{-1} \end{equation*}

The NTCP model of dysgeusia was derived from Sapir *et al.* [19], where *TD*_50_ refers to the tolerance dose leading to 50% complications, with a value of 53.1. *gEUD* represents the generalized equivalent uniform dose. *γ*50 refers to the steepness of the dose complication curve, with a value of 0.973.


(2) \begin{equation*} {NTCP}_{xerostomia}={\left(1+{e}^{-S1}\right)}^{-1} \end{equation*}

The NTCP model of xerostomia was derived from Beetz *et al.* [20], in which *S*1 refers to a coefficient and is calculated as *S*1 = −1.443 + (mean contralateral parotid gland × 0.047) + (baseline xerostomia × 0.720).


(3) \begin{equation*} {NTCP}_{osteoradionecrosis\ of\ the\ mandible}={\left(1+{e}^{-S2}\right)}^{-1} \end{equation*}

The NTCP model of osteoradionecrosis of the mandible was derived from Van *et al*. [21], in which *S*2 refers to a coefficient that is calculated as *S*2 = −9.16 + (D_30%_ mandible × 0.11) + (baseline dental extractions × 0.620). D_30%_ refers to the dose received, which is 30% of the volume of the mandible.

NTCP values were calculated for each patient, and the differences between the both plans (ΔNTCP) were calculated as follows:


(4) \begin{equation*} \Delta NTCP={NTCP}_{VMAT}-{NTCP}_{PSPT} \end{equation*}

PSPT is considered more useful compared with VMAT in the MBA when ΔNTCP exceeds a certain threshold. We used the value adopted by the Dutch National Indication Protocol as a reference for this indicator [22]. Following previous studies, a clinically significant difference in NTCP was considered to exist if the ΔNTCP for grade II complications was ≥10%, if the summed grade II complication was ≥15%, if the ΔNTCP for grade III complications was ≥5% or if the ΔNTCP for grade IV–V complications was ≥2%. For the MBA analysis in this study, patient selection was conducted in the following evaluation order: grade II dysgeusia, grade II xerostomia, summed grade II complication and grade IV osteoradionecrosis of the mandible. Although the endpoints and NTCP models used in this study do not perfectly correspond to those used in the Dutch framework, only NTCP models predicting grade IV or higher osteoradionecrosis are currently available; therefore, the thresholds for each grade were used as references. Based on these criteria, the patients in whom PSPT was considered to have achieved a clinically significant reduction in NTCP were evaluated for each adverse event, and the number of patients who were ultimately eligible was calculated.

### Statistical analysis

The statistical significance of the differences in dose and NTCP metrics between PSPT and VMAT plans was determined using a Wilcoxon signed-rank test. A one-sided test with a significance level of <0.05 was considered significant.

## RESULTS

**Fig. 1:**
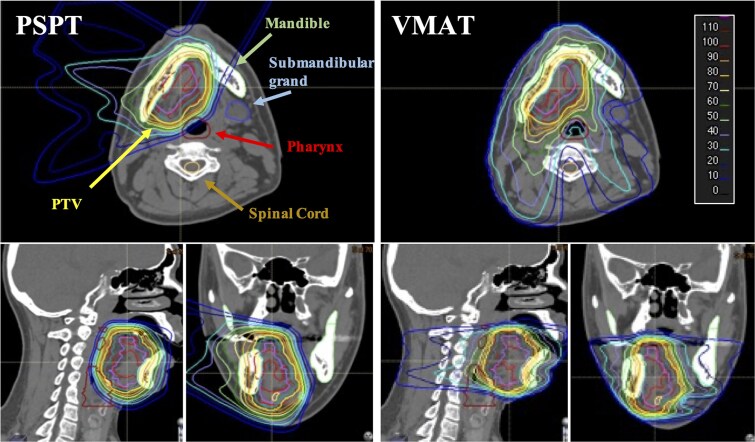
Dose distribution comparison for PSPT (left) and VMAT (right) for tongue cancer. VMAT = volumetric modulated arc therapy, PSPT = passive scattering proton beam therapy.

**Fig. 2:**
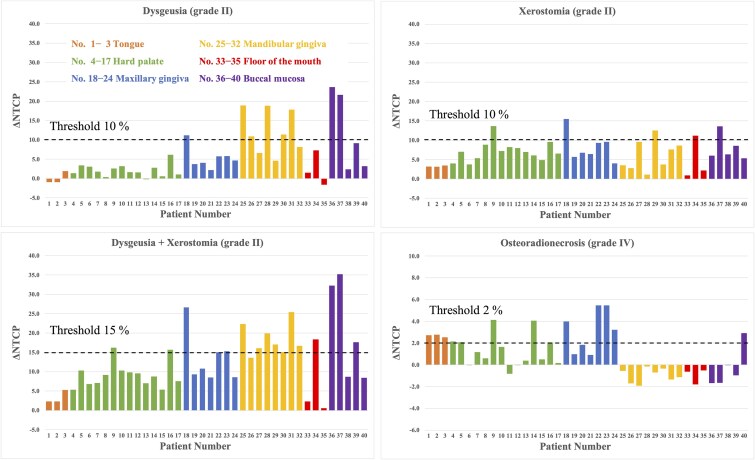
Bar graph showing individual reductions in NTCP. NTCP = normal tissue complication probability.

**Fig. 3:**
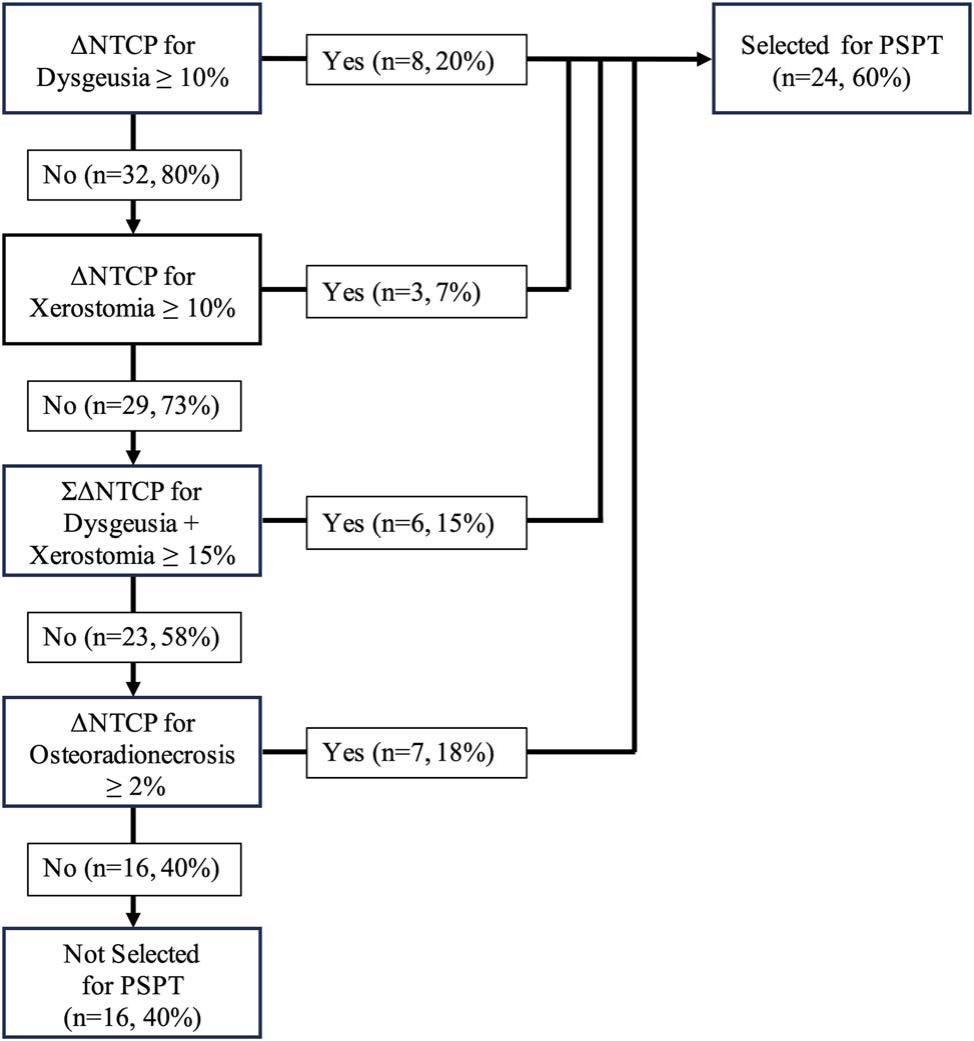
Patient selection using a model-based approach with ΔNTCP criteria. NTCP = normal tissue complication probability, PSPT = passive scattering proton beam therapy.

Patient characteristics are listed in Table 1. Forty patients were enrolled in this study. Various OC subsites were included in this study: 3 cases of tongue cancer, 14 cases of hard palate cancer, 7 cases of maxillary gingival cancer, 8 cases of mandibular gingival cancer, 3 cases of floor of mouth cancer and 5 cases of buccal mucosa cancer. A treatment plan for PSPT and VMAT was created, and a comparison of typical dose distributions is presented in Fig. 1. Although VMAT cannot avoid exposure to low-to-medium doses to the entire oral cavity and contralateral OARs, PSPT allows for localized irradiation to the target. A dose comparison of the OARs is listed in Table 2. PSPT significantly reduced the dose to the spinal cord, pharynx, oral cavity and contralateral salivary glands (*P* < 0.001), whereas no significant difference was observed regarding the maximum dose to the mandible, which is close to the target, and the dose to the ipsilateral salivary gland (*P* > 0.05). In addition, the NTCP values were calculated from the OAR doses. The average NTCP values ​and the evaluation by subsite for dysgeusia, xerostomia and osteoradionecrosis of the mandible are listed in Table 3. Moreover, each ΔNTCP and the number of cases exceeding the MBA threshold are shown in Table 3 and Fig. 2. Regarding the NTCP values for dysgeusia, the average value of PSPT and VMAT for all cases was 11.6 and 17.8%, respectively. The average ΔNTCP for dysgeusia was 5.8%, with a statistically significant difference between PSPT and VMAT (*P* < 0.001). In addition, there were 1, 5 and 2 cases in which the MBA threshold (ΔNTCP ≥10%) was exceeded in the maxillary gingiva, mandibular gingiva and buccal mucosa, respectively. Regarding the NTCP values for xerostomia, the average value for PSPT and VMAT in all cases was 21.8 and 28.4%, respectively. The average ΔNTCP for xerostomia was 6.7%, with a statistically significant difference between PSPT and VMAT (*P* < 0.001). In addition, one case each exceeded the MBA threshold (ΔNTCP ≥10%) in the hard palate, maxillary gingiva, mandibular gingiva, floor of the mouth and buccal mucosa. With respect to NTCP values for osteoradionecrosis of the mandible, the average value of PSPT and VMAT in all cases was 5.7 and 6.4%, respectively. The average ΔNTCP for osteoradionecrosis of the mandible was 0.9%, and there was a statistically significant difference between PSPT and VMAT (*P* < 0.001). Moreover, there were 3, 4, 4 and 1 cases in which the MBA threshold (ΔNTCP ≥2%) was exceeded in the tongue, hard palate, maxillary gingiva and buccal mucosa, respectively. The patient selection results for the MBA using the ΔNTCP criteria are presented in Figs 2 and 3. PSPT was indicated in 24 of the 40 cases (60% of the total), whereas the remaining 16 cases (40% of the total) showed no advantage compared with VMAT.

**Table 1:** Patient characteristics

	Median	Range
Age at PSPT in years	70	19–90
CTV in cm^3^	113.7	26.5–612.3
	*N*	(%)
Gender [male/female]	25/15	(62.5/37.5)
Subsite		
Tongue	3	(7.5)
Hard palate	14	(35.0)
Maxillary gingiva	7	(17.5)
Mandibular gingiva	8	(20.0)
Floor of the mouth	3	(7.5)
Buccal mucosa	5	(12.5)
Histology		
SCC	22	(55.0)
ACC	12	(30.0)
Others	6	(15.0)
T classification		
T3	7	(17.5)
T4A	21	(52.5)
T4B	12	(30.0)
*N* classification		
N0	30	(75.0)
N1	3	(7.5)
N2	7	(17.5)
Stage		
III	7	(17.5)
IVA	21	(52.5)
IVB	12	(30.0)
Baseline xerostomia score		
None	38	(95.0)
A bit	2	(5.0)
Quite a bit	0	(0.0)
A lot	0	(0.0)
Baseline dental extractions		
No	35	(87.5)
Yes	5	(12.5)

PSPT = passive scattering proton beam therapy, CTV = clinical target volume, SCC = squamous cell carcinoma, ACC = adenoid cystic carcinoma.

**Table 2:** Dose volume comparison for target and OARs

Structure	Metric	PSPT		VMAT		*P* value
		Median	Range	Median	Range	
PTV	V_95%_ (%)	94.8	93.0–96.6	95.0	95.0–95.0	0.24
Spinal cord	D_max_ [Gy(RBE)]	2.2	0.0–40.9	17.1	3.4–43.2	<0.001
Pharynx	D_max_ [Gy(RBE)]	70.8	0.4–73.1	72.9	14.7–77.8	<0.001
	D_mean_ [Gy(RBE)]	21.4	0.0–59.5	32.9	4.2–62.3	<0.001
Mandible	D_max_ [Gy(RBE)]	70.6	28.0–75.1	70.5	22.9–70.5	0.32
	D_30%_ [Gy(RBE)]	20.5	0.0–71.5	24.4	0.9–69.9	<0.001
Oral cavity	D_mean_ [Gy(RBE)]	23.1	1.3–63.7	28.6	10.6–67.3	<0.001
Parotid gland (ipsi)	D_mean_ [Gy(RBE)]	28.0	0.0–71.0	21.6	1.7–73.3	0.34
Submandibular gland (ipsi)	D_mean_ [Gy(RBE)]	16.0	0.0–71.5	13.5	0.4–72.9	0.53
Sublingual gland (ipsi)	D_mean_ [Gy(RBE)]	3.0	0.0–71.4	3.7	0.0–72.7	0.21
Parotid gland (contra)	D_mean_ [Gy(RBE)]	0.0	0.0–40.4	7.4	1.1–41.4	<0.001
Submandibular gland (contra)	D_mean_ [Gy(RBE)]	0.1	0.0–68.0	4.0	0.0–68.6	<0.001
Sublingual gland (contra)	D_mean_ [Gy(RBE)]	0.1	0.0–70.8	2.5	0.0–72.0	<0.001

OARs = organs at risks, VMAT = volumetric modulated arc therapy, PSPT = passive scattering proton beam therapy, Avg = average, PTV = planning target volume, ipsi = ipsilateral, contra = contralateral.

**Table 3:** NTCP predictions for different side effect

Side effect/subsite	NTCP_PSPT_ in %	NTCP_VMAT_ in %	ΔNTCP in %	*P* value	Patients with
	Avg (range)	Avg (range)	Avg (range)		ΔNTCP ≥ threshold
Dysgeusia (grade II)					
Total (*n* = 40)	11.6 (0.1–66.9)	17.8 (0.8–71.6)	5.8 (−1.6 to 26.3)	<0.001	8/40 (20.0%)
Tongue (*n* = 3)	18.9 (6.2–23.9)	18.6 (5.3–25.8)	0.1 (−0.9 to 1.9)		0/3 (0.0%)
Hard palate (*n* = 14)	5.2 (0.3–41.8)	7.8 (0.8–42.3)	2.0 (−0.1 to 6.1)		0/14 (0.0%)
Maxillary gingiva (*n* = 7)	3.6 (0.6–10.8)	8.9 (2.3–15.8)	5.3 (2.1 to 11.1)		1/7 (14.3%)
Mandibular gingiva (*n* = 8)	20.0 (1.0–66.9)	32.1 (10.1–71.6)	12.1 (4.6 to 18.8)		5/8 (62.5%)
Floor of the mouth (*n* = 3)	32.6 (3.8–54.3)	34.6 (5.3–52.7)	2.4 (−1.6 to 7.2)		0/3 (0.0%)
Buccal mucosa (*n* = 5)	10.6 (0.1–35.7)	27.1 (2.4–53.9)	12.5 (2.3 to 26.3)		2/5 (40.0%)
Xerostomia (grade II)					
Total (*n* = 40)	21.8 (19.1–61.2)	28.4 (19.9–62.3)	6.7 (0.8 to 15.5)	<0.001	5/40 (12.5%)
Tongue (*n* = 3)	19.1 (19.1–19.1)	22.0 (21.3–22.5)	3.2 (3.2 to 3.4)		0/3 (0.0%)
Hard palate (*n* = 14)	22.0 (19.1–46.5)	29.0 (23.1–53.5)	7.1 (3.8 to 13.6)		1/14 (7.1%)
Maxillary gingiva (*n* = 7)	21.2 (19.1–33.7)	29.9 (23.1–49.1)	8.2 (4.0 to 15.5)		1/7 (14.3%)
Mandibular gingiva (*n* = 8)	25.4 (19.1–61.2)	31.3 (21.8–62.3)	6.1 (1.1 to 12.4)		1/8 (12.5%)
Floor of the mouth (*n* = 3)	19.6 (19.1–20.5)	24.3 (19.9–30.4)	4.7 (0.8 to 11.1)		1/3 (33.3%)
Buccal mucosa (*n* = 5)	19.1 (19.1–19.1)	27.0 (24.4–32.7)	7.9 (5.3 to 13.6)		1/5 (20.0%)
Osteoradionecrosis (grade IV)					
Total (*n* = 40)	5.7 (0.1–22.3)	6.4 (0.1–21.5)	0.9 (−1.9 to 5.4)	<0.001	12/40 (30.0%)
Tongue (*n* = 3)	0.8 (0.6–0.9)	3.2 (3.0–3.4)	2.6 (2.5 to 2.7)		3/3 (100.0%)
Hard palate (*n* = 14)	0.1 (0.1–0.9)	1.4 (0.1–4.4)	1.3 (−0.8 to 4.1)		4/14 (28.6%)
Maxillary gingiva (*n* = 7)	0.1 (0.1–0.3)	3.1 (0.8–5.5)	3.1 (0.9 to 5.4)		4/7 (57.1%)
Mandibular gingiva (*n* = 8)	15.5 (0.7–22.3)	14.3 (0.5–19.3)	−1.0 (−1.9 to −0.1)		0/8 (0.0%)
Floor of the mouth (*n* = 3)	12.1 (0.7–18.2)	11.2 (0.3–17.0)	−1.0 (−1.8 to −0.5)		0/3 (0.0%)
Buccal mucosa (*n* = 5)	12.2 (0.1–22.3)	11.5 (0.1–20.3)	−0.3 (−1.7 to 2.9)		1/5 (20.0%)

NTCP = normal tissue complication probability, VMAT = volumetric modulated arc therapy, PSPT = passive scattering proton beam therapy, Avg = average.

## DISCUSSION

In this study, several NTCP models were used to convert the dose difference between PSPT and VMAT into clinically relevant benefits in 40 OC patients. During the MBA analysis, PBT was selected if the expected reduction in the NTCP value met the predefined eligibility criteria, and 24 of 40 patients were eligible for PSPT. The results indicated that PSPT was superior to VMAT in reducing NTCP for dysgeusia and xerostomia in almost all cases. Using PSPT, it is possible to reduce the dose around the PTV and to the contralateral OARs, whereas with VMAT, it is impossible, in principle, to avoid irradiating the OARs. Therefore, PSPT is more advantageous than VMAT in terms of reducing the dose to the contralateral salivary glands and oral cavity region (Table 2). In Table 3, the risk difference for dysgeusia and xerostomia also exhibited a significant reduction of NTCP in PSPT (*P* < 0.001). Many other studies have indicated that the use of PBT can reduce the risk of xerostomia, which is consistent with the findings presented in the present study [14–16, 23]. Conversely, a value of 10% was used as the threshold for ΔNTCP for grade II late adverse events in the present study; however, when compared with the individual ΔNTCP, there were not many cases exceeding this threshold (Fig. 2 and Table 3). When evaluated in conjunction with a risk assessment of multiple complications (summed ΔNTCP >15%), PSPT was found to be applicable in a greater number of cases (Fig. 2). To assess the NTCP for grade IV osteoradionecrosis of the mandible, the dose to the mandible was determined. As listed in Table 2, in OC cases, the target and mandible are often very close to one another, and the maximum dose to the mandible is not different from that of VMAT (*P* = 0.32). Conversely, low-to-medium doses may be readily reduced, and there is a significant difference in the metric of D_30%_ metric used to calculate NTCP (*P* < 0.001). Although the NTCP value was particularly high in tongue cancer, this study represented a simulation that prioritized target coverage, and in actual clinical practice, it will be necessary to appropriately adjust the dose to the mandible to prevent osteoradionecrosis of the mandible. As shown in Table 3 and Fig. 2, although the absolute difference in ΔNTCP was small, it showed a statistically significant difference (*P* < 0.001), and many cases exceeded the threshold for indication (ΔNTCP ≥2%).

The MBA evaluation revealed that PSPT was superior in 60% of the cases overall, indicating the advantage of selecting PBT in OC cases; however, the location of the primary tumor varied depending on the subsite. Table 3 shows the NTCP for the three complications evaluated by subsite, and the expected reduction in NTCP value was different. Thus, the magnitude of the benefit from PSPT varied according to the subsite, which can be primarily explained by anatomical differences and the relative proximity between the tumor and OARs. Tumors located in subsites such as the tongue and the floor of the mouth are surrounded by highly radiosensitive tissues, including the pharynx and salivary glands; therefore, the steep dose gradients achievable with PBT can enhance normal tissue sparing. In contrast, for subsites where the distance between the target and adjacent OARs is relatively greater, the resulting benefit was smaller. In addition, it is important to use the appropriate NTCP model for each patient. In the present study, three models were used: xerostomia, dysgeusia and osteoradionecrosis of the mandible; however, depending on the subsite, the risk assessment of dysphagia or blindness may be appropriate [24, 25]. Several NTCP models are used in MBA, but careful consideration must be given to the choice. This evidence-based treatment decision by MBA plays an important role because it shows the benefits of PBT to patients with rare tumors in which long-term treatment results are not available. While further expansion of its use is expected, treatment planning requires time-consuming and considerable resources. Recently, there have been active studies into contouring and treatment planning automation using artificial intelligence. The adoption of these technologies may simplify the work and shorten the time from the first consultation to the first treatment [26, 27].

One limitation of this study is that intensity modulated proton therapy (IMPT) using the pencil beam scanning (PBS) technique was not evaluated. Recently, PBS delivery technology has become clinically widespread because of the development of irradiation technology. Compared with PSPT, which has a limitation of reducing the dose to OARs in cases with complex target shapes, IMPT can provide an even better target coverage and dose reduction to OARs [28, 29]; however, because IMPT is a recently developed irradiation method, there is a lack of cases with long-term follow-up. Because most reliable data on PBT for OC are based on conventional methods, we attempted to examine it using PSPT as an initial step. In the present study, good results were observed, even with PSPT; however, future studies are required to determine the extent to which IMPT can improve these results. Furthermore, because this study was conducted at a single institution, its generalizability may be limited by institution-specific factors, including contouring methods, treatment planning policies and system-specific characteristics. Therefore, multi-institutional validation is therefore warranted. In this study, the potential clinical benefit of PBT was estimated using an NTCP model. However, our analysis was not directly compared with toxicity data actually observed in OC patients, partly because some cases have not yet accumulated sufficient follow-up to allow a direct comparison between the predicted estimates and the actual clinical outcomes. Therefore, the extent to which the clinical benefit of PBT is accurately reflected remains to be clarified. Future clinical and external validation studies will be important to confirm whether the findings of this study are consistent with actual clinical outcomes. In conclusion, OC patients were selected for PBT based on MBA. Compared with VMAT, the risk of expected late adverse events may be significantly reduced with PBT, and 60% of the OC patients were considered eligible for PBT in the MBA.

## PRESENTATION AT A CONFERENCE

This study was presented at the 37th Annual Meeting of the Japanese Society for Radiation Oncology.
